# Supplementary material for: Divergent midbrain circuits orchestrate escape and freezing responses to looming stimuli in mice
Source: Nat Commun. 2018 Mar 26;9:1232. doi: 10.1038/s41467-018-03580-7 (PMC5964329; doi:10.1038/s41467-018-03580-7)
Supplement: Supplementary file 1 — Supplementary Information-PDF(PDF 3573 kb) [file 41467_2018_3580_MOESM1_ESM.pdf]

Supplementary Figures and Tables

Divergent midbrain circuits orchestrate escape and freezing responses to looming stimuli in mice

Authors: Congping Shang, Zijun Chen, Aixue Liu, Yang Li, Jiajing Zhang, Fei Yan, Baole Qu, Yaning Zhang, Weixiu Liu, Zhihui Liu, Xiaofei Guo, Dapeng Li, Yi Wang, Peng Cao

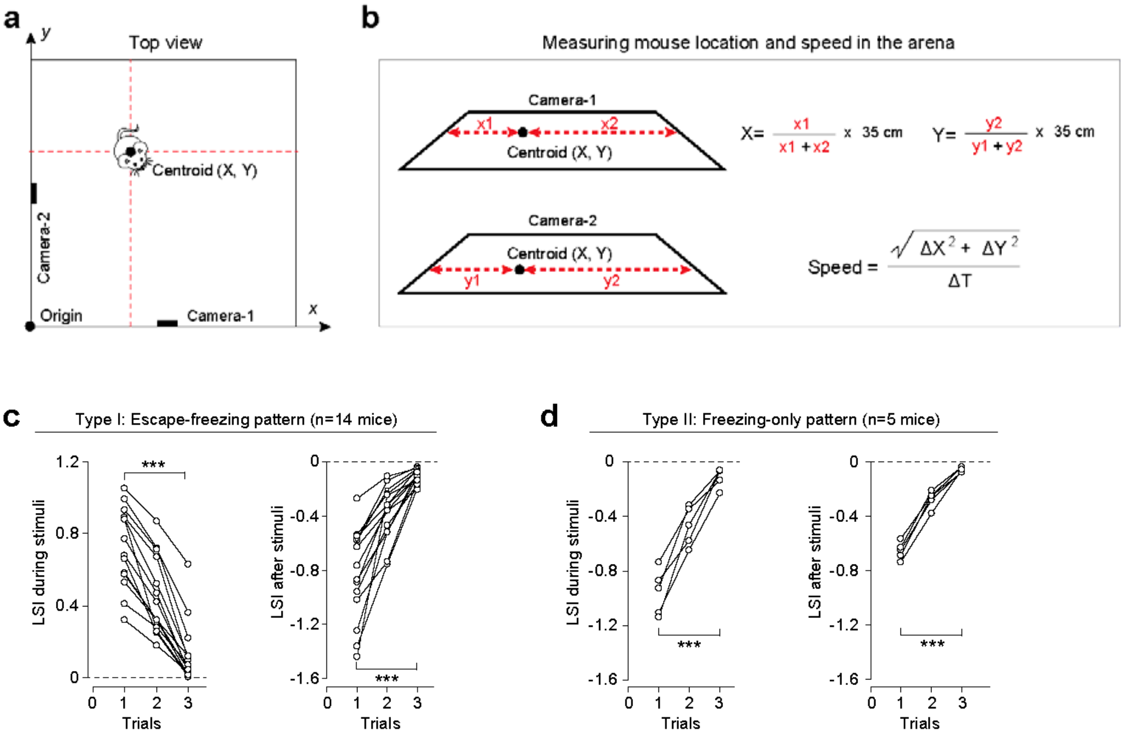

Supplementary Figure 1 Quantitative analyses of mouse defensive behaviors triggered by looming visual stimuli. (a) Schematic diagram showing the centroid of a mouse in the *x*-*y* coordinate of the behavioral arena. The mouse locomotion was monitored by two orthogonally positioned cameras (camera-1, camera-2). **(b)** Schematic diagram and mathematical formulae to measure mouse Location (X, Y) and Speed in the arena. For more information, see Methods (session "Behavioral tests"). (**c**) LSI $_{\text{during stimuli}}$ (*left*) and LSI $_{\text{after stimuli}}$ (*right*) of the same mice (n=14 mice) with type I defensive behavioral pattern to three trials of looming visual stimuli (1 trial per minute). (**d**) LSI $_{\text{during stimuli}}$ (*left*) and LSI $_{\text{after stimuli}}$ (*right*) of the same mice (n=5 mice) with type II defensive behavioral pattern to three trials of looming visual stimuli (1 trial per minute). Data points in (c, d) indicate each mouse. Statistical analysis in (c, d) was Student's *t* test (*** $P<0.001$).

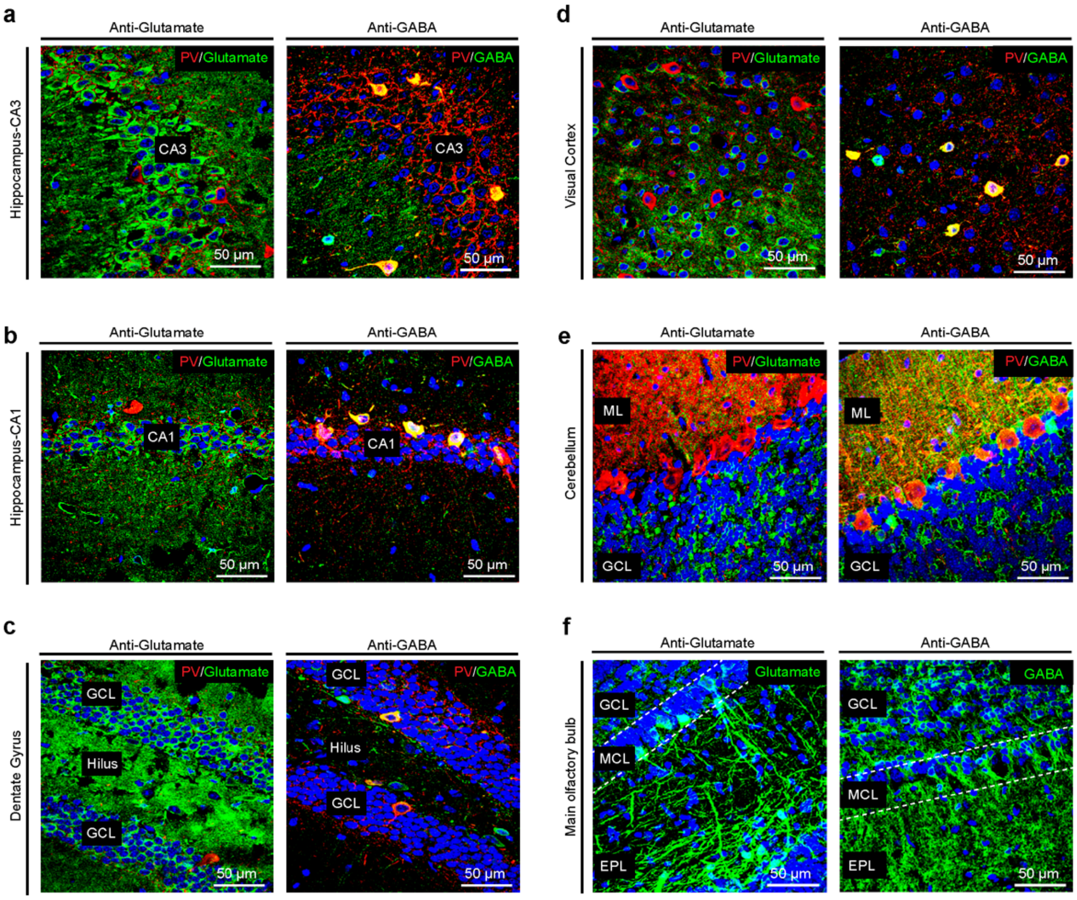

Supplementary Figure 2 Anti-glutamate and anti-GABA antibodies validated by immunohistochemistry. (**a - e**) Micrographs showing immunoreactivity of glutamate vs. PV (left column), and that of GABA vs. PV (right column) in the hippocampal CA3 (a), CA1 (b), dentate gyrus (c), visual cortex (d) and cerebellum (e). In the hippocampus (a-c), CA3/CA1 pyramidal neurons (a, b) and granule cells in the dentate gyrus (c) are labeled by anti-glutamate antibody but not by anti-GABA antibody, whereas PV^+ GABAergic neurons are labeled by anti-GABA antibody but not anti-glutamate antibody. In the visual cortex (d), cortical GABAergic PV^+ neurons are labeled by anti-GABA antibody but not by anti-glutamate antibody. In the cerebellum (e), PV^+ GABAergic Purkinje cells are labeled by anti-GABA antibody but not by anti-glutamate antibody. (**f**) In the olfactory bulb, anti-glutamate antibody labeled mitral cells in the mitral cell layer (MCL) and tufted cells in the external plexiform layer (EPL), without labeling granule cells in the granule cell layer (GCL). By contrast, anti-GABA antibody labeled massive granule cells in the GCL. These data suggest that the anti-glutamate and anti-GABA antibodies can reliably label glutamatergic and GABAergic neurons in mouse brain. Scale bars are indicated in the graphs.

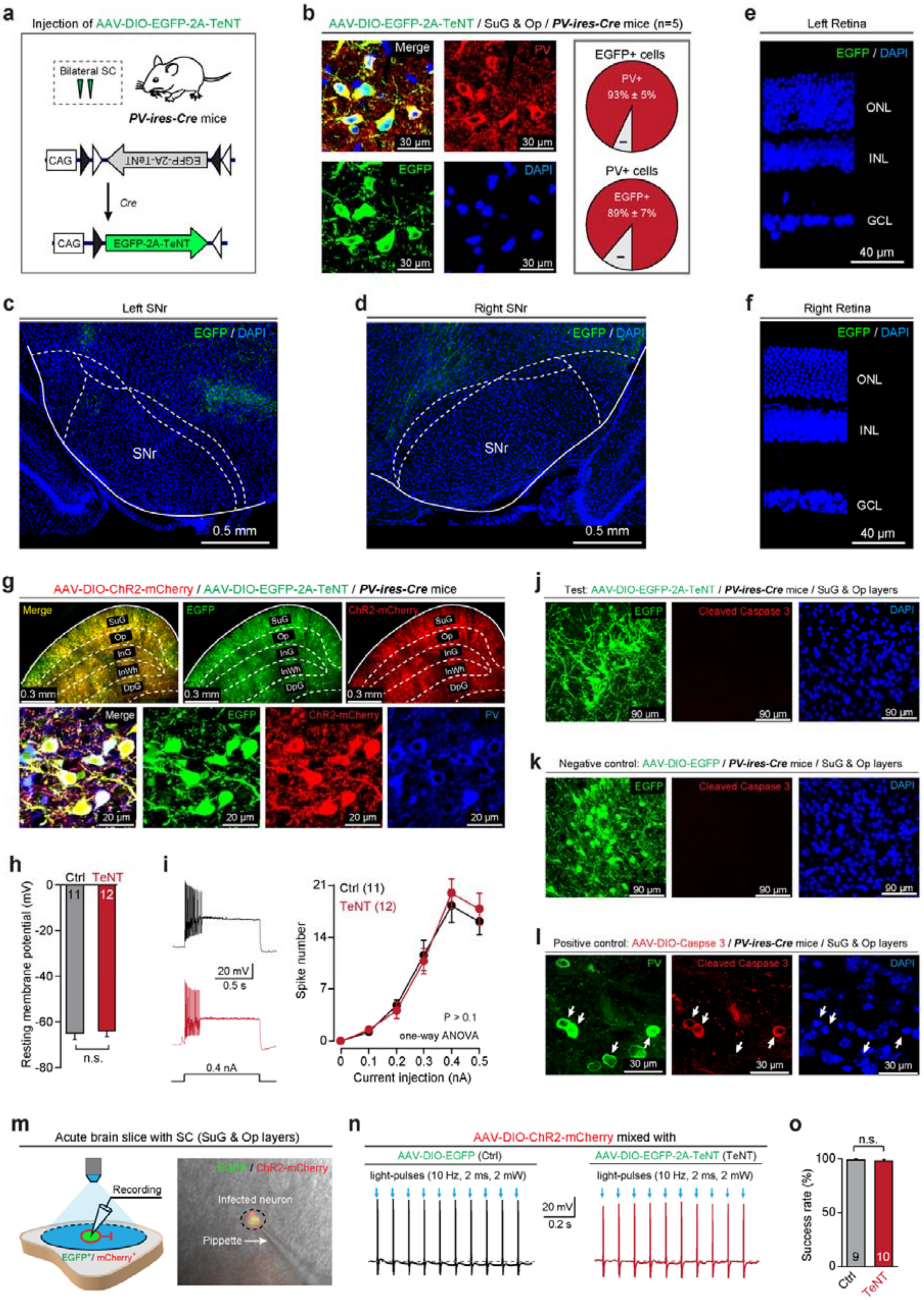
a
Injection of AAV-DIO-EGFP-2A-TeNT
Bilateral SC
PV-ires-Cre mice
CAG
EGFP-2A-TeNT
Cre
b
AAV-DIO-EGFP-2A-TeNT / SuG & Op / PV-ires-Cre mice (n=5)
Merge
PV
EGFP
DAPI
30 μm
EGFP+ cells
PV+
93% ± 5%
PV+ cells
EGFP+
89% ± 7%
e
Left Retina
EGFP / DAPI
ONL
INL
GCL
40 μm
c
Left SNr
SNr
0.5 mm
d
Right SNr
f
Right Retina
g
AAV-DIO-ChR2-mCherry / AAV-DIO-EGFP-2A-TeNT / PV-ires-Cre mice
ChR2-mCherry
SuG
Op
InG
InWh
DpG
0.3 mm
20 μm
j
Test: AAV-DIO-EGFP-2A-TeNT / PV-ires-Cre mice / SuG & Op layers
Cleaved Caspase 3
90 μm
k
Negative control: AAV-DIO-EGFP / PV-ires-Cre mice / SuG & Op layers
l
Positive control: AAV-DIO-Caspse 3 / PV-ires-Cre mice / SuG & Op layers
h
Ctrl
TeNT
11
12
Resting membrane potential (mV)
n.s.
i
20 mV
0.5 s
0.4 nA
Ctrl (11)
TeNT (12)
Spike number
P > 0.1
one-way ANOVA
Current injection (nA)
m
Acute brain slice with SC (SuG & Op layers)
Recording
EGFP+/ mCherry+
EGFP / ChR2-mCherry
Infected neuron
Pippette
n
AAV-DIO-ChR2-mCherry mixed with
AAV-DIO-EGFP (Ctrl)
AAV-DIO-EGFP-2A-TeNT (TeNT)
light-pulses (10 Hz, 2 ms, 2 mW)
20 mV
0.2 s
o
Success rate (%)
n.s.
9
10

Supplementary Figure 3 SC PV+ neurons are essential for visually triggered dimorphic defensive behaviors. (a) Schematic diagram showing injection of AAV-DIO-EGFP-2A-TeNT into the SC of *PV-ires-Cre* mouse line. **(b)** Example micrographs (left) and quantitative analyses (right) showing PV^+ neurons in the SuG and Op layers were reliably labeled by EGFP after AAV-DIO-EGFP-2A-TeNT injection into the SC of *PV-ires-Cre* mice (n=5). For cell counting strategy, see Methods and Supplementary Table 1. **(c, d)** Example brain tissue sections of left (c) and right SNr (d) showing no retrogradely infected cell bodies were labeled in the SNr. **(e, f)** Example sections of left (e) and right retina (f) showing no retrogradely infected cell bodies were labeled in the retina. **(g)** Coronal section of the SC (top) and the micrographs (bottom) showing SC PV^+ neurons co-labeled by EGFP and ChR2-mCherry after unilateral injection of AAV-DIO-EGFP-2A-TeNT and AAV-DIO-ChR2-mCherry mixture into the SC of *PV-ires-Cre* mice. **(h)** Quantitative analyses of the effect of TeNT expression on resting membrane potential of SC PV^+ neurons three weeks after AAV injection. **(i)** Quantitative analyses of the effect of TeNT expression on depolarization-induced action potential firing three weeks after AAV injection. **(j-l)** Example micrographs showing no apoptosis, as indicated by cleaved caspase 3, was observed three months after AAV-DIO-EGFP-2A-TeNT injection into the SC of PV-ires-Cre mice (j). Note (k) is a negative control for (j). In positive control experiment (l), AAV-DIO-Capase3 induced robust Cleaved Caspase 3 signal in SC PV+ neurons, suggesting the antibody of Cleaved Caspase 3 works well. (**m**) Schematic diagram (*left*) and example microscopic image (right) showing recording of light-evoked spiking activity from SC PV^+ neurons infected by AAV-DIO-EGFP-2A-TeNT and AAV-DIO-ChR2-mCherry. The recorded neurons are co-infected and fluorescently positive for both EGFP and mCherry. (**n**) Example traces showing that light-pulse train (473 nm, 10 Hz, 2 ms, 2 mW) reliably evoked phase-locked spiking activity from SC PV^+ neurons expressing ChR2-mCherry, EGFP and TeNT. (**o**) Comparison of success rate to evoke action potential firing from ChR2-mCherry$^+$ neurons with (TeNT) or without TeNT (Ctrl). Data in (h, I, o) are means ± SEM (error bars). Numbers of cells are indicated in the bars. Statistical analysis was Student's *t* test (h and o, n.s. P > 0.1) and Two-way ANOVA (i).

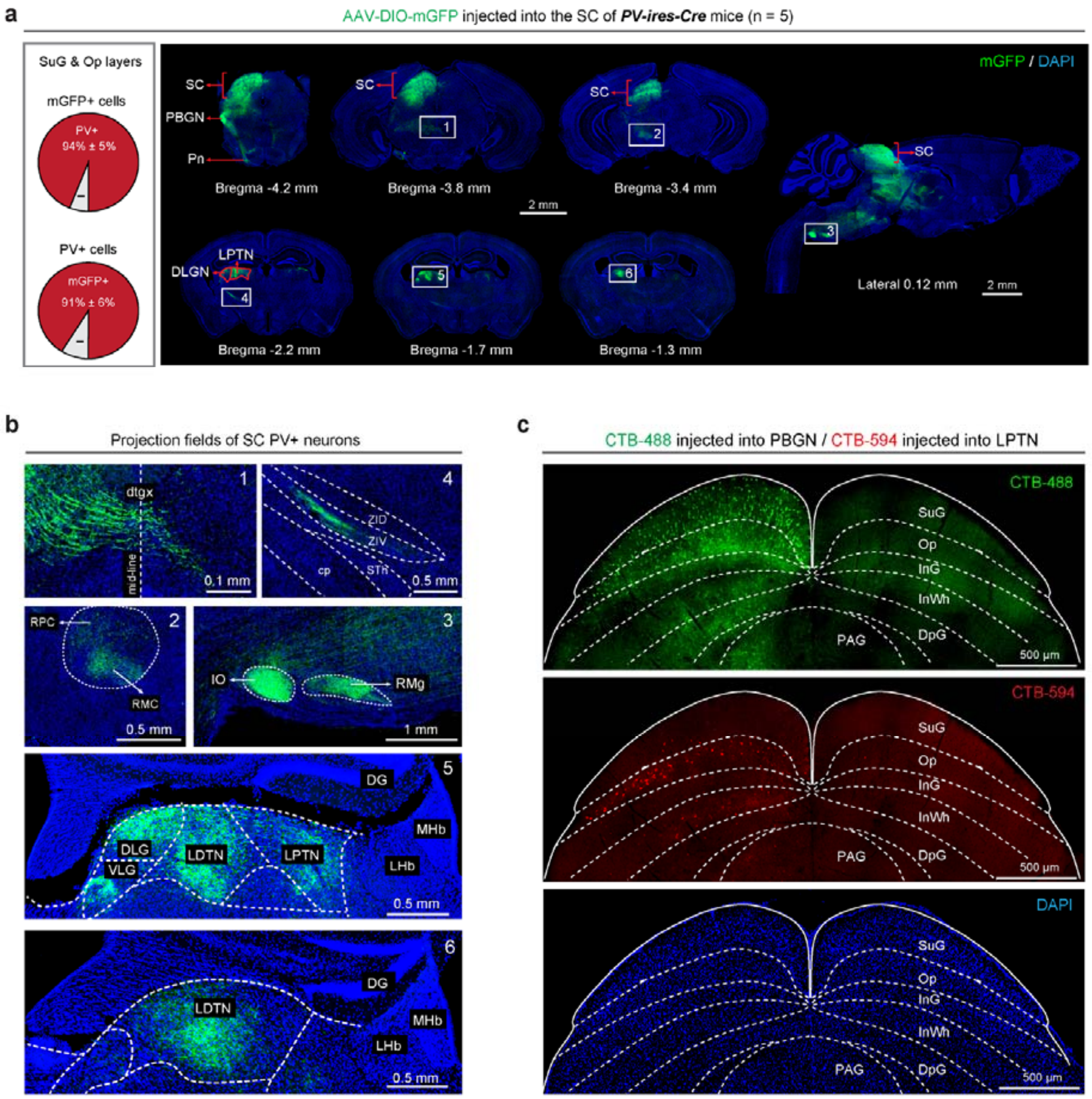

Supplementary Figure 4 Brain-wide mapping of projections from SC PV$^+$ neurons. **(a)** *Left*, quantitative analyses showing PV$^+$ neurons in the SuG and Op layers were reliably labeled by mGFP after AAV-DIO-mGFP injection into the SC of *PV-ires-Cre* mice. For cell counting strategy, see Methods and Supplementary Table 1. *Right*, coronal and sagittal sections showing SC PV$^+$ neurons labeled by mGFP project extensively to diverse brain regions. The cropped regions (1-6) are shown in (b). **(b)** Six micrographs of the magnified regions cropped in (a) showing the projection fields of the SC PV$^+$ neurons. Note axons of some SC PV$^+$ neurons crossed the brain midline at dtgx (region 1). Axons of some SC PV$^+$ neurons projected ipsilaterally to RPC/RMC (region 2), IO (region 3), RMg (region 3), ZIV (region 4), LPTN (region 5), DLG (region 5), VLG (region 5) and

LDTN (region 5 and 6). RPC, red nucleus, parvicellular part; RMC, red nucleus, magnocellular part; IO, inferior olive; RMg, Raphe magnus nucleus; ZIV, ventral zona incerta; LPTN, lateral posterior thalamic nucleus; DLG, dorsal lateral geniculate nucleus; VLG, ventral lateral geniculate nucleus; LDTN, lateral dorsal thalamic nucleus. **(c)** Three single-channel micrographs showing an example coronal section of the SC with retrogradely labeled cells projecting to the PBGN (CTB-488^{+}, green) and LPTN (CTB-594^{+}, red). Scale bars are indicated in each panel. Data in (a, left) are means ± SEM (error bars).

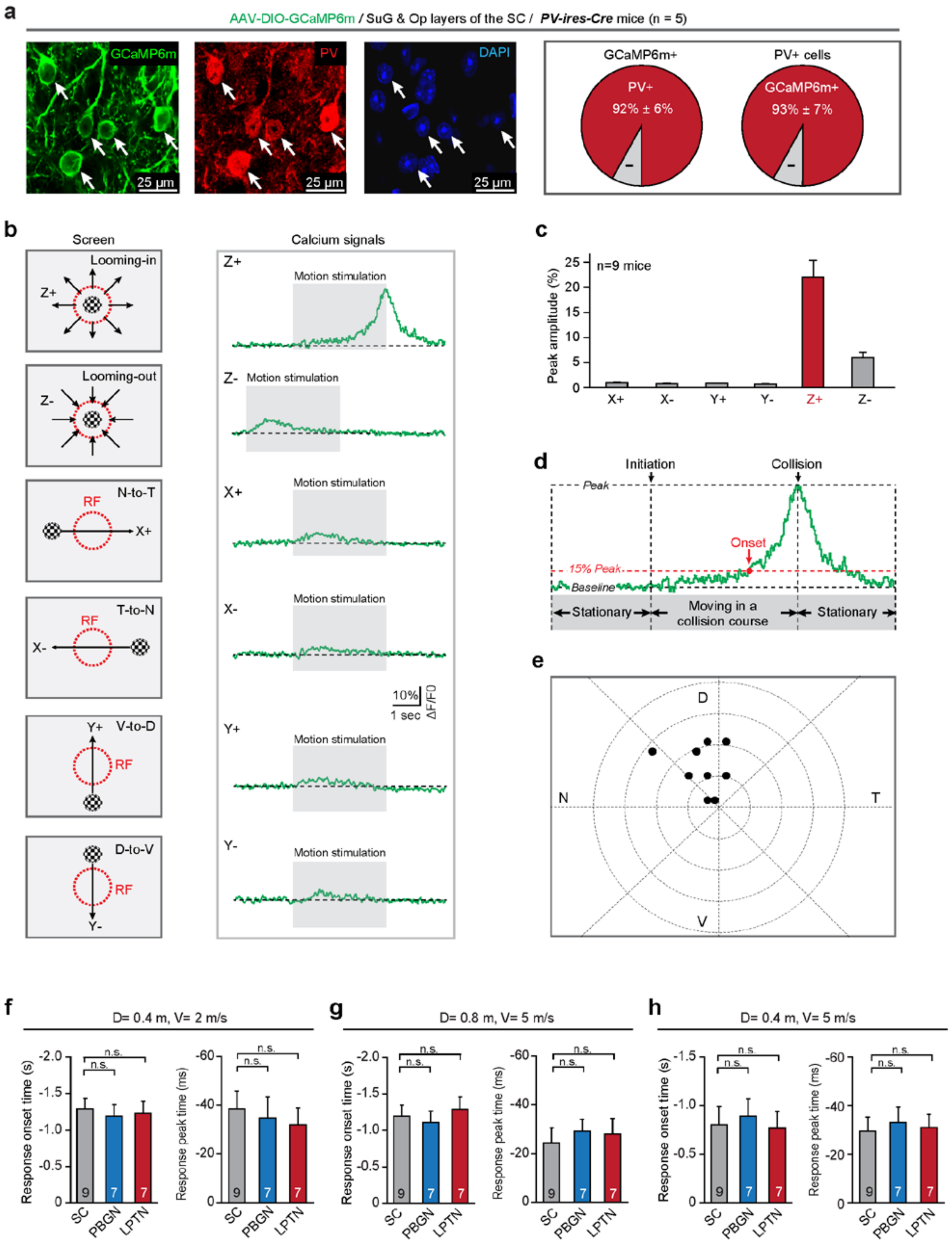
a
AAV-DIO-GCaMP6m / SuG & Op layers of the SC / PV-ires-Cre mice (n = 5)
GCaMP6m
PV
DAPI
25 μm
GCaMP6m+
PV+ cells
PV+
92% ± 6%
GCaMP6m+
93% ± 7%
b
Screen
Looming-in
Looming-out
N-to-T
T-to-N
V-to-D
D-to-V
RF
Calcium signals
Motion stimulation
10%
1 sec
ΔF/F0
c
n=9 mice
Peak amplitude (%)
X+
X-
Y+
Y-
Z+
Z-
d
Initiation
Collision
Peak
Onset
15% Peak
Baseline
Stationary
Moving in a collision course
Stationary
e
D
N
T
V
f
D= 0.4 m, V= 2 m/s
g
D= 0.8 m, V= 5 m/s
h
D= 0.4 m, V= 5 m/s
Response onset time (s)
Response peak time (ms)
n.s.
SC
PBGN
LPTN

Supplementary Figure 5 Recording of visually evoked Ca^{2+} transients from SC PV^+ neurons expressing GCaMP6m. (**a**) Single-channel micrographs (left) and quantitative analyses (right) showing PV^+ neurons in the SuG and Op layers were reliably labeled by GCaMP6m after AAV-DIO-GCaMP6m injection into the SC of *PV-ires-Cre* mice. Arrows indicate PV^+ neurons labeled by GCaMP6m. For cell counting strategy, see Methods and Supplementary Table 1. (**b**) Schematic diagram showing the motion patterns of the soccer ball on the screen (left column) and example traces of Ca^{2+} transients (right column) recorded from a group of PV^+ neurons in the medial SC in response to these visual stimuli. (**c**) Averaged peak amplitude ($\Delta F/F_0$) of Ca^{2+} responses of SC PV^+ neurons to the soccer ball moving in six directions (n = 9 mice). (**d**) Schematic diagram showing how to measure the onset of Ca^{2+} responses of SC PV^+ neurons to looming visual stimuli. The response onset is defined as the time point when the Ca^{2+} signal reaches 15% of peak amplitude in response to looming visual stimuli. (**e**) Distribution of receptive field centers of recorded SC PV^+ neurons in the 9 mice. (**f**, **g**, **h**) Response onset time (*left*) and response peak time (*right*) of SC PV^+ neurons or their axon terminals in the PBGN or LPTN to the virtual looming soccer ball with different combinations of diameter and velocity. Data in (a, c, f, g, h) are means ± SEM (error bars). Numbers of mice are indicated in the graphs.

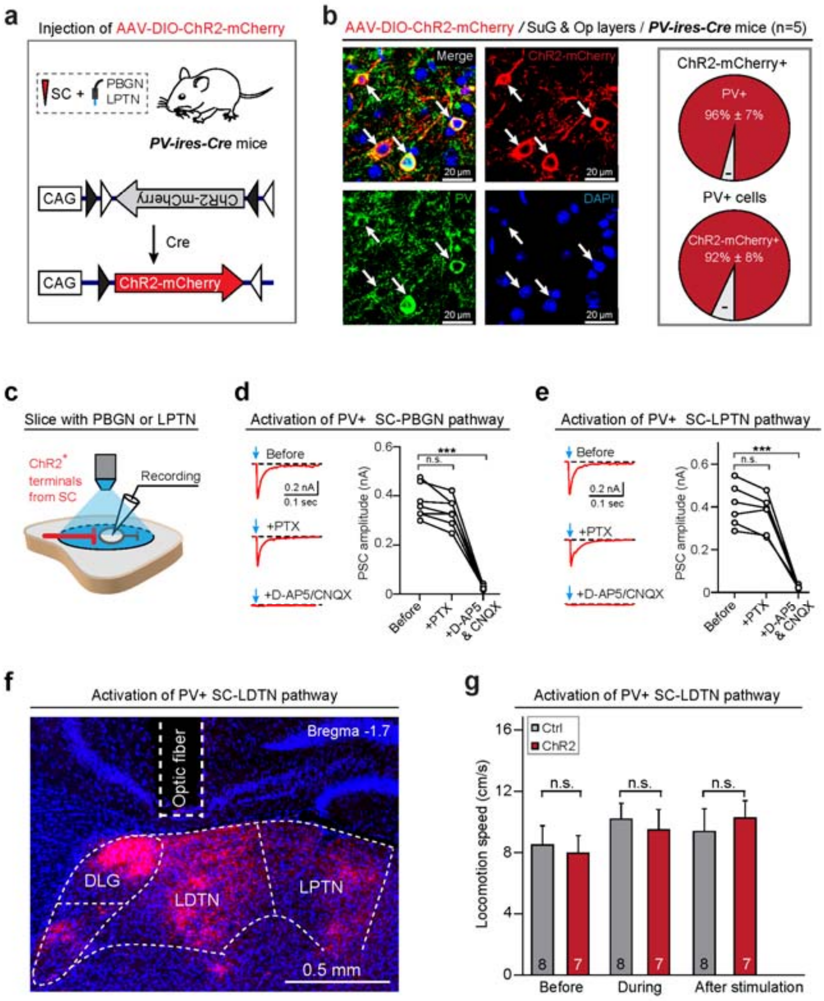

Supplementary Figure 6 Activation of PV$^+$ SC-LPTN and PV$^+$ SC-PBGN pathways mimicked dimorphic defensive behavioral patterns. (**a**) Schematic diagram showing injection of AAV-DIO-ChR2-mCherry into the SC of *PV-ires-Cre* mice followed by optic fiber implantation above the PBGN or LPTN. (**b**) Example micrographs (left) and quantitative analyses (right) showing PV$^+$ neurons in the SuG and Op layers were reliably labeled by ChR2-mCherry after AAV-DIO-ChR2-mCherry injection into the SC of *PV-ires-Cre* mice. For cell counting strategy, see Methods and Supplementary Table 1. (**c**) Schematic diagram of slice physiology showing whole-cell recording of light-evoked PSCs (473 nm, 1 ms, 20 mW) from PBGN or LPTN neurons innervated by ChR2$^+$ axon terminals from SC PV$^+$ neurons. (**d, e**) Effects of PTX and D-AP5/CNQX on light-evoked PSCs recorded from PBGN (d) and LPTN neurons (e). (**f, g**) Example coronal section with optic fiber track above LDTN (f) and average speed before, during, and after activation of PV$^+$ SC-LDTN pathway (g). Data in (b, g) are means ± SEM (error bars). Numbers of mice are indicated in the graphs. Statistical analysis was Student's *t* test (***P < 0.001; n.s. P > 0.1).

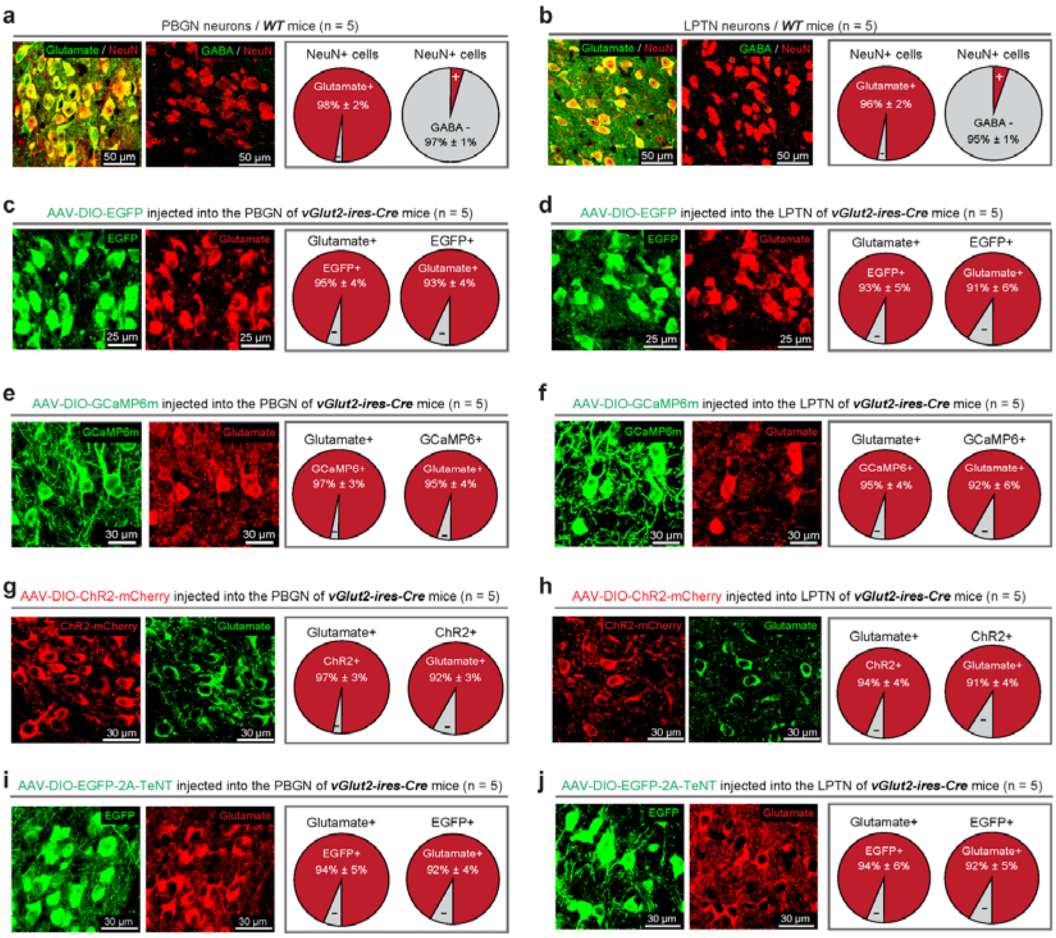

Supplementary Figure 7 Analyses of neuronal types in the PBGN and LPTN. (**a, b**) Example micrographs (left) and quantitative analyses (right) showing neurons ($NeuN^+$) in the PBGN (a) and LPTN (b) of WT mice are predominantly $glutamate^+$ but not $GABA^+$. (**c, d**) Example micrographs (left) and quantitative analyses (right) showing $glutamate^+$ neurons in the PBGN (c) and LPTN (d) were reliably labeled by EGFP after AAV-DIO-EGFP injection into the PBGN and LPTN of *vGlut2-ires-Cre* mice. **(e, f)** Example micrographs (left) and quantitative analyses (right) showing $glutamate^+$ neurons in the PBGN (e) and LPTN (f) were reliably labeled by GCaMP6m after AAV-DIO-GCaMP6m injection into the PBGN and LPTN of *vGlut2-ires-Cre* mice. **(g, h)** Example micrographs (left) and quantitative analyses (right) showing $glutamate^+$ neurons in the PBGN (g) and LPTN (h) were reliably labeled by ChR2-mCherry after AAV-DIO-ChR2-mCherry injection into the PBGN and LPTN of *vGlut2-ires-Cre* mice. **(i, j)** Example micrographs (left) and quantitative analyses (right) showing $glutamate^+$ neurons in the PBGN (i) and LPTN (j) were reliably labeled by EGFP after AAV-DIO-EGFP-2A-TeNT injection into the PBGN and LPTN of *vGlut2-ires-Cre* mice. Data in (a-j) are means ± SEM (error bars). Numbers of mice are indicated in the graphs. For cell counting strategy, see Methods and Supplementary Table 1.

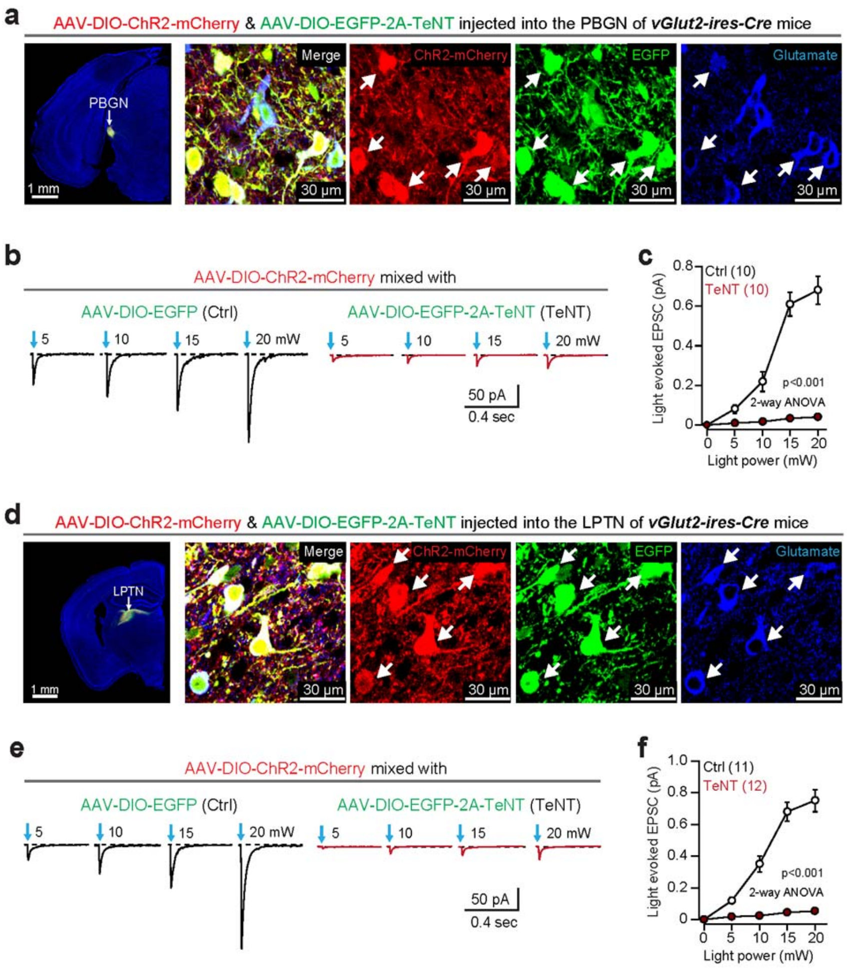

Supplementary Figure 8 Synaptic inactivation of glutamatergic neurons in the PBGN and LPTN. (**a, d**) A mixture of AAV-DIO-ChR2-mCherry and AAV-DIO-EGFP-2A-TeNT was injected into the PBGN (a, left) and LPTN (d, left) of *vGlut2-ires-Cre* mice, resulting in the co-expression of ChR2-mCherry and EGFP in glutamate^{+} neurons in the PBGN (a, right) and LPTN (d, right). (**b, c, e, f**) Example traces (b, e) and input-output curves (c, f) of light-evoked excitatory PSC from ChR2-mCherry-negative neurons in the PBGN (b, c) and LPTN (e, f). Data in (c, f) are means ± SEM (error bars). Numbers of cells are indicated in the graphs. Statistical analysis was Two-way ANOVA. Scale bars are indicated in the graphs. Arrows indicate glutamatergic neurons labeled by both ChR2-mCherry and EGFP.

Supplementary Table 1 Summary of Cell Counting Strategy

Brain region	SuG & Op layers of the SC	PBGN	LPTN
Section type	Coronal	Coronal	Coronal
Section Range	Bregma (-3.28 to -4.48)	Bregma (-4.16 to -4.36)	Bregma (-1.82 to -2.80)
Total collection	Approximately 30 sections	Approximately 5 sections	Approximately 25 sections
Sampling	6 sections evenly<br>spaced by 200 μm	All the 5 sections	5 sections evenly<br>spaced by 200 μm
Histological Analyses	PV vs. Glutamate<br>PV vs. GABA<br>***WT*** mice (n = 5)	Glutamate vs. NeuN<br>GABA vs. NeuN<br>***WT*** mice (n = 5)	Glutamate vs. NeuN<br>GABA vs. NeuN<br>***WT*** mice (n = 5)
	PV vs. mGFP<br>***PV-ires-Cre*** mice (n = 5)<br>inject AAV-DIO-mGFP	Glutamate vs. EGFP<br>***vGlut2-ires-Cre*** mice (n = 5)<br>inject AAV-DIO-EGFP	Glutamate vs. EGFP<br>***vGlut2-ires-Cre*** mice (n = 5)<br>inject AAV-DIO-EGFP
	PV vs. GCaMP6<br>***PV-ires-Cre*** mice (n = 5)<br>Inject AAV-DIO-GCaMP6	Glutamate vs. GCaMP6<br>***vGlut2-ires-Cre*** mice (n = 5)<br>inject AAV-DIO-GCaMP6	Glutamate vs. GCaMP6<br>***vGlut2-ires-Cre*** mice (n = 5)<br>inject AAV-DIO-GCaMP6
	PV vs. ChR2-mCherry<br>***PV-ires-Cre*** mice (n = 5)<br>inject AAV-DIO-ChR2mCherry	Glutamate vs. ChR2-mCherry<br>***vGlut2-ires-Cre*** mice (n = 5)<br>inject AAV-DIO-ChR2mCherry	Glutamate vs.<br>ChR2-mCherry<br>***vGlut2-ires-Cre*** mice (n = 5)<br>inject<br>AAV-DIO-ChR2-mCherry
	PV vs. EGFP-2A-TeNT<br>***PV-ires-Cre*** mice (n = 5)<br>Inject AAV-DIO-EGFP-2A-TeNT	Glutamate vs. EGFP-2A-TeNT<br>***vGlut2-ires-Cre*** mice (n = 5)<br>inject<br>AAV-DIO-EGFP-2A-TeNT	Glutamate vs.<br>EGFP-2A-TeNT<br>***vGlut2-ires-Cre*** mice (n = 5)<br>inject<br>AAV-DIO-EGFP-2A-TeNT
	PV vs. CTB-488 and -594<br>CTB-488 injected into the PBGN<br>CTB-594 injected into the LPTN<br>of ***WT*** mice (n = 3)	N.A.	N.A.

Supplementary Table 2 AAV injection, optic fiber implantation & CTB injection

Brain Regions	SC	PBGN	LPTN
AAV-DIO-EGFP-2A-TeNT	Bilateral injections ***(Figure 2)***<br>SC: B -3.8, L ± 0.5, D -1.0	Bilateral injections ***(Figure 8)***<br>PBGN: B -4.25, L ± 1.95, D -2.60	Bilateral injections ***(Figure 8)***<br>LPTN: B -2.06, L ± 1.35, D -2.40
AAV-DIO-mGFP	Unilateral injection ***(Figure 3)***<br>SC: B -3.8, L ± 0.5, D -1.0	N.A.<br>N.A.	N.A.<br>N.A.
AAV-DIO-EGFP	N.A.<br>N.A.	Unilateral injection ***(Figure 6)***<br>PBGN: B -4.25, L +1.95, D -2.60	Unilateral injection ***(Figure 6)***<br>LPTN: B -2.06, L +1.35, D -2.40
AAV-DIO-GCaMP6	Unilateral injection ***(Figure 4)***<br>SC: B-3.80, L+0.50, D-1.00<br><br>Ipsilateral implantation<br>SC: B-3.80, L+0.50, D -0.50<br>PBGN: B-4.25, L+1.95, D-2.50<br>LPTN: B-2.06, L+1.35, D-2.20	Unilateral injection ***(Figure 6)***<br>PBGN: B -4.25, L +1.95, D -2.60<br><br>Ipsilateral implantation<br>PBGN: B -4.25, L +1.95, D -2.50	Unilateral injection ***(Figure 6)***<br>LPTN: B -2.06, L +1.35, D -2.40<br><br>Ipsilateral implantation<br>LPTN: B -2.06, L +1.35, D -2.20
AAV-DIO-ChR2-mCherry	Unilateral injection ***(Figure 5)***<br>SC: B -3.80, L +0.50, D -1.00<br><br>Ipsilateral implantation<br>PBGN:B-4.25, L+1.95, D-2.50<br>LPTN:B-2.06, L+1.35, D-2.20<br>LDTN:B-1.20, L+1.00, D-2.00	Unilateral injection ***(Figure 7)***<br>PBGN: AP -4.25, L +1.95, D -2.60<br><br>Ipsilateral implantation<br>PBGN: AP -4.25, L +1.95, D -2.50	Unilateral injection ***(Figure 7)***<br>LPTN: B -2.06, L +1.35, D -2.40<br><br>Ipsilateral implantation<br>LPTN: B -2.06, L +1.35, D -2.20
AAV-DIO-EGFP-2A -NaChBac	N.A.<br>N.A.	Bilateral injections ***(Figure 9)***<br>PBGN: B -4.25, L ± 1.95, D -2.60	Bilateral injections ***(Figure 9)***<br>LPTN: B-2.06, L±1.35, D-2.40
CTB-488	N.A.<br>N.A.	Unilateral injection ***(Figure 3)***<br>PBGN: AP-4.25,L +1.95, D -2.60	N.A.<br>N.A.
CTB-594	N.A.<br>N.A.	N.A.<br>N.A.	Unilateral injection ***(Figure 3)***<br>LPTN: B-2.06, L+1.35, D-2.40

Supplementary Table 3: Summary of reagents and mouse lines

Antibodies		
Anti-EGFP	Abcam	ab290
Anti-parvalbumin	Millipore	MAB1572
Anti-glutamate	Sigma	G6642
Anti-GABA	Sigma	A2052
Anti-NeuN	Millipore	MAB377
Mouse lines		
PV-ires-Cre	JAX Mice	Stock No: 008069
vGlut2-ires-Cre	JAX Mice	Stock No: 028863
Chemicals and tracers		
D-AP5	Tocris	Cat. No. 0106
CNQX	Tocris	Cat. No. 0190
Picrotoxin	Tocris	Cat. No. 1128
TTX	Tocris	Cat. No. 1078
4-AP	Sigma	Cat. No. 275875
CTB-594	Thermo-Fisher	Cat. No. C22842
CTB-488	Thermo-Fisher	Cat. No. C34775
AAV vectors		
AAV-DIO-EGFP-2A-TeNT	Stanford Vector Core	N.A.
AAV-DIO-EGFP	Stanford Vector Core	N.A.
AAV-DIO-mGFP	Stanford Vector Core	N.A.
AAV-DIO-ChR2-mCherry	Stanford Vector Core	N.A.
AAV-DIO-mCherry	Stanford Vector Core	N.A.
AAV-DIO-GCaMP6m	Stanford Vector Core	N.A.
AAV-DIO-EGFP-2A-N aChBac	Stanford Vector Core	N.A.
