# Supplementary material for: Divergent midbrain circuits orchestrate escape and freezing responses to looming stimuli in mice
Source: Nat Commun. 2018 Mar 26;9:1232. doi: 10.1038/s41467-018-03580-7 (PMC5964329; doi:10.1038/s41467-018-03580-7)
Supplement: Supplementary file 3 — Description of Additional Supplementary Information(PDF 55 kb) [file 41467_2018_3580_MOESM3_ESM.pdf]

Description of Additional Supplementary Files

File Name: Supplementary Movie 1
Description: Example of type I defensive behavior in wild-type mice.

File Name: Supplementary Movie 2
Description: Example of type II defensive behavior in wild-type mice.

File Name: Supplementary Movie 3
Description: Effects of synaptic inactivation of SC PV+ neurons on visually triggered defensive behaviour.

File Name: Supplementary Movie 4
Description: Defensive behaviors triggered by optogenetic activation of PV+ SC-PBGN and SC-LPTN pathways.

File Name: Supplementary Movie 5
Description: Fiber photometry recording of glutamatergic PBGN neurons during defensive behaviors in a freely moving mouse.

File Name: Supplementary Movie 6
Description: Fiber photometry recording of glutamatergic LPTN neurons during defensive behaviors in a freely moving mouse.

File Name: Supplementary Movie 7
Description: Defensive behaviors triggered by optogenetic activation of glutamatergic PBGN neurons and LPTN neurons.

File Name: Supplementary Movie 8
Description: Effects of synaptic inactivation of glutamatergic PBGN neurons on visually triggered defensive behavior.

File Name: Supplementary Movie 9
Description: Effects of synaptic inactivation of glutamatergic LPTN neurons on visually triggered defensive behavior.
